# Editorial: Breaking barriers, bridging gaps: UN World AIDS Day 2023

**DOI:** 10.3389/fpubh.2025.1728931

**Published:** 2025-11-10

**Authors:** Hailay Abrha Gesesew, John Shearer Lambert

**Affiliations:** 1 Research Centre for Public Health, Equity and Human Flourishing (PHEHF), Adelaide, SA, Australia; 2 College of Health Sciences, Mekelle University, Mekelle, Tigray, Ethiopia; 3 University College Dublin, Dublin, Ireland

**Keywords:** UN Day, AIDS, stigma, HIV diagnosis, HIV prevention, HIV incidence, health systems, HIV delivery

## Background

World AIDS Day is a moment to reflect on both the achievements and the ongoing challenges of the global HIV response—including the decline in new HIV infections and HIV-related deaths, the expansion of antiretroviral therapy (ART) coverage, and the growing concern of drug resistance. At the same time, it reminds us of the remaining obstacles: persistent new infections, unequal progress among populations such as those in conflict-affected or vulnerable settings, and the continuing need for sustained funding and political commitment.

As part of this exploration and in recognition of the United Nations World AIDS Day 2023, by 22 of December 2023, a Research Topic of the Frontiers in Public Health entitled “*Breaking barriers, bridging gaps: UN World AIDS Day 2023*,” was opened and a team of scholars has handled the editorial work as guest editors to facilitate the timely peer-review and publication of relevant manuscripts from multiple studies. The Research Topic brings together global scholarship and local experience to reflect on the progress and ongoing challenges in the HIV response.

A total of 19 manuscripts were submitted of which seven were rejected. Twelve manuscripts from 46 contributing authors from Ethiopia (four), Uganda (one), Zambia (one), Kenya (one), Namibia (one), South Africa (one), Thailand (one), and China (two), represent Africa and Asia, two most affected contents by the HIV epidemic, were published between 22 December 2023 and 22 June 2024. Population in the studies included adults living with HIV, adolescents living with HIV and their caregivers, other high-risk populations and older people living with HIV/AIDS.

Together, these works advance our understanding of the multifaceted determinants of HIV care and the innovative approaches being adopted to close gaps in health systems, psychosocial support, and community engagement. By October 2025, the Research Topic achieved 30,000 views and downloads. In this Research Topic, key thematic areas were discussed including but not limited to:

1. Health system readiness and differentiated service delivery (DSD)—Research from Africa, for example, Kaonga et al. from Zambia identified that less than 50% of the facilities had all indicators of availability and readiness, respectively. These findings underscore the importance of capacity building, infrastructure investment, and decentralized care in improving retention and treatment adherence. Community-based DSD models evaluated in Ethiopia (e.g. Merid et al.) and Zambia (e.g. Kaonga et al.) illustrate how such innovations enhance convenience and continuity, particularly for populations with limited access to traditional health facilities.
2. Treatment outcomes, viral suppression, and survival—Research from Ethiopia, Namibia, and China focuses on time to viral load suppression, rapid ART initiation, and determinants of time to death. For example, Chen et al. from China revealed a significant association between rapid ART initiation and reduced risk of viral failure; and Abeje et al. from Ethiopia found that the change in increasing in viral load was high during the latter follow-up period compared to the beginning of the follow-up period.
3. Psychosocial dimensions and stigma—Some contributions explore the complex psychosocial terrain of living with HIV. For example, analyses of body mass index (BMI) and perceived stigma by Desta et al. found that patients with non-adherent to HAART and poor social support were more likely to suffer from HIV-related perceived stigma. Studies on mental phenotypes among individuals with well-controlled HIV by Rubin et al. and on psychosocial and mental health challenges of perinatally infected adolescents by Wanjala et al. bring attention to an often-overlooked aspect of the epidemic—the cognitive, emotional, and developmental impacts that persist even in the era of effective treatment. Work on unsuppressed adolescents and their caregivers further illuminates how familial and community support are essential to sustained engagement in care.
4. Prevention, retention, and the power of hope—Prevention continues to evolve, as reflected in research examining pre-exposure prophylaxis (PrEP) uptake and retention among high-risk populations by Rayanakorn et al.. These insights reinforce the value of differentiated prevention strategies to meet the needs of diverse communities. Finally, a study on hope levels among older people living with HIV by Yu et al. from China captures an important, humanistic dimension of the response—reminding us that the goal of HIV care is not only biological control but also psychological resilience, social inclusion, and dignity in aging.

## Conclusion

We hope that our Edited Research Topic provides multidimensional evidence to enhance understanding of the factors affecting HIV care and highlights effective approaches to close gaps within the HIV care system.

